# Exploring the prevalence of anabolic steroid use among men and women resistance training practitioners after the COVID-19 pandemic

**DOI:** 10.1186/s12889-024-18292-5

**Published:** 2024-03-13

**Authors:** Rastegar Hoseini, Zahra Hoseini

**Affiliations:** Department of Exercise Physiology, Faculty of Sport Sciences, Razi University, Kermanshah, P.O. Box. 6714414971, Iran

**Keywords:** COVID-19, Resistance training, AS, Young adults, Awareness campaigns

## Abstract

**Background:**

The COVID-19 pandemic has had a significant impact on individual health and fitness routines globally. Resistance training, in particular, has become increasingly popular among men and women looking to maintain or improve their physical fitness during the pandemic. However, using Anabolic Steroids (AS) for performance enhancement in resistance training has known adverse effects. Thus, this study aimed to explore the prevalence of AS use among men and women resistance training practitioners after the COVID-19 pandemic.

**Methods:**

A cross-sectional survey was conducted among 3,603 resistance training practitioners (1,855 men and 1,748 women) in various geographical locations impacted by COVID-19. The participants were asked to complete self-administered questionnaires, which included questions regarding demographic information, training habits, and current or prior usage of AS. The data were analyzed using SPSS statistical software and the chi-square method, with a significance level of (*P* < 0.05).

**Results:**

A total of 3603 men and women resistance training practitioners completed the survey. In the study, 53.05% of men and 41.99% of women used anabolic and androgenic steroids. Of those men who used steroids, 29.47% used Testosterone, while 31.20% of women used Winstrol. Additionally, 50.30% of men used steroids via injection, while 49.05% of women used them orally. According to the study, 49.99% of the participants had 6 to 12 months of experience with resistance training, and 64.25% of them underwent three training sessions per week. The analysis using the χ2 test did not reveal any significant difference between men and women in terms of duration of bodybuilding, frequency per week, and engagement in other activities.

**Conclusion:**

This study shows that a significant proportion of men and women resistance training practitioners used AS, particularly among young adults with limited training experience. Thus, there is a need for targeted education and awareness campaigns to address the hazards of AS use and promote healthy training habits during the COVID-19 pandemic.

## Introduction

The coronavirus pandemic has caused significant disruption to the daily activities of individuals across the world [1]. One of the areas of life that has been significantly affected is physical exercise [2]. With the closure of gyms and other sports facilities and restrictions on outdoor exercise, resistance training practitioners have been forced to adapt to new methods of training to maintain their fitness levels [3]. This disruption to training habits may have had an impact on Anabolic Steroid (AS) usage among men and women resistance training practitioners [4]. AS are synthetic substances designed to mimic the effects of natural testosterone in the body [5]. These substances have numerous applications, including medical treatment for hormonal imbalances and muscle-wasting diseases. However, their abuse in the fitness industry, particularly among bodybuilders and other resistance training practitioners, has become widespread, primarily due to their performance-enhancing effects.

Several previous studies have explored the prevalence of AS consumption in various populations, including athletes, bodybuilders, and fitness enthusiasts [6, 7]. These studies have demonstrated that the use of these substances is not limited to men and is consumed among women as well. According to a meta-analysis studying a wide range of samples, such as students, university students, resistance training practitioners, and the general public, the global prevalence of AS consumption was estimated at approximately 3.3% [8]. About Iran, a prevalence of 0.3% AS consumption among the adult population [6, 9]. This percentage increased to 36.66% in 2020, which was primarily investigated among men aged between 18 and 34 years [10]. Furthermore, the prevalence of AS consumption varies widely across different countries, with rates of up to 25% reported in some cases [11, 12]. Recent studies have raised concerns that the Coronavirus Disease 2019 (COVID-19) COVID-19 pandemic might have led to an increased prevalence of AS consumption in the resistance training community [4, 13]. The pandemic response measures have forced many people to adjust to new, home-based training methods, with limited availability of gym and fitness facilities. This shift in training patterns may have led to increased use or abuse of AS among resistance training practitioners. However, the novelty of this study lies in its focus on resistance training practitioners, the examination of anabolic steroid use after the COVID-19 pandemic, and the inclusion of both men and women in the study population. To date, no research has explored the prevalence of AS consumption among resistance trainers after the COVID-19 pandemic in the Iranian population. Despite the growing popularity of resistance training during the pandemic, there is limited research specifically examining the prevalence of AS use among individuals engaging in resistance training. This research gap is crucial as it allows us to understand the extent of AS use and its associated risks within this specific population. By addressing this research gap, valuable insights can be provided into the prevalence of AS use and its potential implications for the health and well-being of resistance training practitioners. This study aims to address the research gap by exploring the prevalence of AS use among men and women resistance training practitioners after the COVID-19 pandemic. The novelty of this study lies in its focus on a specific population and its potential to provide valuable insights into AS use within the resistance training community. By linking the research gap to the goal of the article, the existing literature aims to be contributed to and awareness of the hazards of AS use while fostering healthy training habits during the COVID-19 pandemic aims to be promoted. Thus, this study aimed to investigate the prevalence of AS consumption among men and women resistance training practitioners after the COVID-19 pandemic.

## Methods

### Study design and population

The survey was conducted in Kermanshah, Iran, a city with an approximate population of 1 million. The survey obtained information on the number and location of gyms in Kermanshah from the Regional Council of Physical Education in the city between May and July 2023. A total of 356 fitness centers that offered resistance training were identified, out of which 286 centers were included in the study. With a confidence interval of 95% and assuming a p = q = 50% probability, a total of 100 resistance training centers were calculated, with an error margin of 7.9%, and used to estimate the population of resistance training practitioners in the city. The gyms were selected randomly and systematically from the five administrative regions of the city, based on the proportion of the number of gyms in each region. The gym management was contacted and explained about the study before obtaining their consent. Individuals aged 18 years and above, training for resistance exercise during morning, afternoon, or night hours were identified in each center. On average, 568 resistance training practitioners were identified per gym. A total number of 4,198 individuals were selected proportionately from each gym based on the number of resistance training practitioners, with a sampling error of 1.25% and a confidence interval of 95%. After screening out incomplete responses, 3603 individuals (1,855 men and 1,748 women) were included in the final analyses. At the commencement of the questionnaires, the participants were provided with information regarding the objectives of the study, and the confidential handling of data, and participants completed the consent form. Also, all educated participants and the legal guardians of illiterate participants were asked to complete the written informed consent at the beginning of the study. The study was conducted in adherence to the seventh and current modification (World Medical Association, 2013) of the Declaration of Helsinki. All experimental protocols were approved by the Committee of Research in Public Sports Board, Kermanshah, Iran.

### Data collection

A self-administered questionnaire, consisting of 32 questions, was devised through a scholarly literature review of relevant articles [14, 15]. The Questionnaire included the following variables: gender, age, profession, marital status, schooling, socioeconomic status, practice time of resistance training, duration, and purpose of training, nutritional monitoring, use of supplements, and use of AS. The questionnaire underwent a validation process, which determined its clarity, content, and construct indices. The questionnaire’s construction and content were evaluated and validated by professional health practitioners, while the clarity aspect was reviewed by individuals sharing the same traits, including class, age, and lifestyle of the intended research population. A pilot study was conducted to assess the questionnaire’s feasibility for use among the target populace.

To standardize the approach and application of the questionnaire, a pre-training session was conducted with the researchers. Following the pre-training, a pilot study with 40 individuals was carried out at the Kani Gym, which was not included in the survey data. Data collection was conducted throughout the working day by researchers positioned at the entrance of the gym and dressed in uniform to be easily identified. To approach participants, they were explained the research purpose, either at the beginning or end of their workout. Participants who agreed to participate in the study signed an informed consent form. The researchers provided clarification for any queries or ambiguities related to the questionnaire before allowing the participants to complete the form independently, without interference.

### Statistical analysis

Statistical analysis in this study was performed using SPSS statistical software (version 21; SPSS Inc., Chicago, IL, USA) with a significance level of *P* < 0.05. The normality of distribution was assessed with the Kolmogorov-Smirnov test. Both descriptive statistics, including mean, standard deviation, and percentage, and deductive statistics the Chi-square method, were utilized for analysis.

## Results

A total of 3,603 (1,855 men and 1,748 women) resistance training practitioners from various regions participated in the survey. A total of (number) participants took part in this study, of which 46% were aged between 18 and 29 years old (46.15% men and 45.08% women), 34.08% were aged between 30 and 44 years old (34.17% men and 33.98% women), 14.13% were aged between 45 and 59 years old (14.33% men and 13.90% women), and 6.16% were aged over 60 years old (5.34% men and 7.04% women). Also, 27.59% were single (30.02% men and 25.06% women), and 72.41% were married (69.98% men and 74.94% women). Furthermore, 0.72% of the participants were illiterate (0.81% men and 0.63% women), while 19.18% had a bachelor’s degree (18.01% men and 20.42% women). The majority of the participants, 80.10%, were university-educated (18.18% men and 78.95% women). Regarding employment status, 44.93% of the participants were employed (68.46% men and 19.97% women), 30.01% were enrolled as students (27.01% men and 33.18% women), and 25.06% were unemployed (4.53% men and 46.85% women). Only a small proportion of the sample, 3.99%, reported being smokers (4.96% men and 2.98% women), while 13.60% of the participants were hospitalized due to COVID-19 (15.94% men and 12.08% women) (Table 1).

**Table 1:** Characteristics and gender differences of resistance training practitioners

Variable	Total n (%)	Men n (%)	Women n (%)	Statistics
Age	3603	1855 (51.5%)	1748(48.5%)	0.053
18 to 29 years	1644(45.63%)	856 (46.15%)	788(45.08%)	0.341
30 to 44 years	1228(34.08%)	634 (34.17%)	594((33.98%)	0.892
45 to 59 years	509(14.13%)	266(14.34%)	243(13.90%)	0.734
≥ 60 years	222(6.16%)	99(5.34%)	123(7.04%)	0.433
Marital status				
Single	995(27.59%)	557(30.02%)	438(25.06%)	0.056
Married	2608(72.41%)	1298(69.98%)	1310(74.94%)	0.055
Educational level				
Illiterate	26(0.72%)	15(0.81%)	11(0.63%)	0.087
Undergraduate	691(19.18%)	334(18.01%)	357(20.42%)	0.096
College	2886(80.10%)	1506(81.18%)	1380(78.95%)	0.197
Occupational status				
Employed	1619(44.93%)	1270(68.46%)	349(19.97%)	0.001*
Student	1081(30.01%)	501(27.01%)	580(33.18%)	0.049*
Unemployed	903(25.06%)	84(4.53%)	819(46.85%)	0.001*
Smoking				
Yes	144(3.99%)	92(4.96%)	52(2.98%)	0.143
No	3459(96.01%)	1763(95.04%)	1696(97.02%)	0.114
Hospitalization for COVID infection				
Yes	490(13.60%)	279(15.04%)	211(12.08%)	0.087
No	3113(86.40)	1576(84.96%)	1537(87.92%)	0.067

Data analysis was done by the χ2 test

*: Significantly different, comparing men and women

The χ2 test was conducted to examine potential gender differences for all of these variables. The results demonstrated that employment status was the only variable with a statistically significant gender difference, with a higher proportion of men being employed compared to women (*p* < 0.05). No significant differences were found in the distribution of other variables based on gender (Table 1).

In this study, 49.99% of the participants had 6 to 12 months of experience with resistance training, and 64.25% of them underwent three training sessions per week. The results of analysis using the χ2 test revealed no significant difference in the duration of bodybuilding, frequency per week, and engagement in other activities between men and women. However, a significant difference in the purpose of performing resistance exercises was found, with 51.37% of men attending the gym for hypertrophy and 55.94% of women attending for weight loss. These findings suggest that men and women exhibit similar patterns of engagement in resistance training, but their motivations for doing so may differ (Table 2).

**Table 2:** Patterns of participation and motivations for resistance training in men and women

Variable	Total n (%)	Men n (%)	Women n (%)	Statistics
Duration of bodybuilding				
< 6 months	1411(39.16%)	742(40%)	669(38.27%)	0.341
≥ 6 months and < 1 year	1801(49.99%)	927(49.97%)	874(50%)	0.536
≥ 1 year and < 3 years	298(8.27%)	123(6.63%)	175(10.01%)	0.066
≥ 3 years	93(2.58%)	63(3.40%)	30(1.72%)	0.135
Frequency per week				
2 times	664(18.43%)	349(18.81%)	315(18.02%)	0.774
3 times	2315(64.25%)	1179(63.56%)	1136(64.99%)	0.657
4 times	387(10.74%)	202(10.89%)	185(10.58%)	0.867
5 or more times	237(6.58%)	125(6.74%)	112(6.41%)	0.894
Objective				
Hypertrophy	1155(32.06%)	953(51.37%)	202(11.56%)	0.001*
Weight loss	1627(45.16%)	649(34.99%)	978(55.94%)	0.001*
Resistance	381(10.57%)	135(7.28%)	246(14.07%)	0.024*
Strength	319(8.85%)	65(3.50%)	254(14.53%)	0.001*
Other	121(3.36%)	53(2.86%)	68(3.90%)	0.583
Practicing another activity				
Yes	2069(57.42%)	1056(53.96%)	1013(57.95%)	0.098
No	1534(42.58%)	799(43.07%)	735(42.05%)	0.472

Data analysis was done by the χ2 test

*: Significantly different, comparing men and women

Table 3 presents the results showing that 53.05% of men and 41.99% of women used anabolic and androgenic steroids, with consumption methods differing between genders; 50.30% of men used it via injection, while 49.05% of women used it orally. The results of the χ2 test demonstrated a significant difference in the amount and consumption method of anabolic and androgenic steroid use between men and women. Furthermore, it was found that Testosterone was used by 29.47% of men, while Winstrol was used by 31.20% of women. These findings provide insight into gender-based differences in the use of anabolic and androgenic steroids and suggest that gender-specific strategies may be necessary to address this practice.

**Table 3:** Gender-based differences in anabolic and androgenic steroid use

Variable	Total n (%)	Men n (%)	Women n (%)	Statistics
Anabolic-Androgenic Steroid				
Yes	1718(47.68%)	984(53.05%)	734(41.99%)	0.001*
No	1885(52.32%)	871(46.95%)	1014(58.01%)	0.001*
Consumption Method				
Injection	688(40.05%)	495(50.30%)	193(26.29%)	0.001*
Oral Intake	714(41.56%)	354(35.97%)	360(49.05%)	0.001*
Gel and Cream	183(10.65)	88(8.95%)	95(12.94%)	0.048*
All	133(7.74%)	47(4.78%)	86(11.72%)	0.031*
Type				
Testosterone	396(23.05%)	290(29.47%)	106(14.44%)	0.001*
Dianabol	382(22.23%)	231(23.48%)	151(20.57%)	0.156
Anadrol	325(18.92%)	146(14.84%)	179(24.39%)	0.011*
Trenbolone	66(3.84%)	43(4.37%)	23(3.13%)	0.253
Turinabol	148(8.62%)	102(10.36%)	46(6.27%)	0.029*
Winstrol	401(23.34%)	172(17.48%)	229(31.20%)	0.001*

Data analysis was done by the χ2 test

*: Significantly different, comparing men and women

## Discussion

Resistance training is a popular form of exercise that has gained significant attention in recent years due to its numerous health benefits. The current study aims to investigate the exploring the prevalence of AS use among men and women resistance training practitioners after the COVID-19 pandemic. The results of the present study revealed a sample of 3,603 individuals, with approximately equal representation of men and women (51.42% versus 48.58%, respectively). The age distribution of participants showed that resistance training is popular among young adults, with 46% of participants aged between 18 and 29 years old. The originality of our study lies in its comprehensive analysis of the characteristics and gender differences of resistance training practitioners from various regions. This age range was nearly uniformly split between men and women; this finding is significant as it indicates that resistance training is equally popular among both genders, particularly among young adults, with 46% of participants aged between 18 and 29 years old. The findings of the present study indicate that the majority of participants were university-educated, which is consistent with previous research demonstrating that a higher level of education is associated with higher participation in exercise and sports [16, 17]. Additionally, the results showed that the majority of participants were married, which suggests that resistance training may be a popular form of exercise for those with responsibilities such as marriage and children. In terms of employment status, these results suggest that there is a gender difference, with a higher proportion of men being employed compared to women. This finding is consistent with previous research demonstrating that men are more likely to be employed than women [18], and may reflect societal norms and gender roles. Finally, this study revealed a low prevalence of smoking among resistance training practitioners, which is encouraging given the detrimental health effects of smoking. However, a relatively high rate of hospitalization due to COVID-19 was found among the sample, which could be attributed to increased exposure to the virus in fitness facilities. This emphasizes the importance of implementing and promoting preventive measures to mitigate the risk of COVID-19 transmission in fitness facilities Overall, this study contributes to a better understanding of the characteristics and gender differences of resistance training practitioners from various regions. These findings suggest that resistance training is popular among both genders, particularly among young adults, and can be practiced by individuals with diverse educational and marital backgrounds. This is significant as it broadens our understanding of the demographic profile of resistance training practitioners. However, future research should investigate the motivations and expectations of resistance training practitioners, as well as the factors that influence how this form of exercise is adopted and maintained over time.

Also, the results of this study showed that a high percentage of participants had between 6 and 12 months of resistance training experience, and the majority underwent three weekly training sessions. Furthermore, these results showed no significant differences in the length of bodybuilding, frequency per week, and engagement in other activities between genders. These findings suggest that men and women exhibit similar exercise habits in resistance training. However, a significant difference in motivations between genders was found. The gym was attended by over half of the men (51.37%) for hypertrophy, while over half of the women (55.94%) attended for weight loss. Thus, these findings indicate that the motivations behind resistance training may differ between genders. It is worth noting that despite these differences in motivations, both men and women seem to have an equal level of engagement in resistance training. These findings have important implications for resistance training interventions. For example, hypertrophy may be less of a motivator for women in resistance training, while emphasizing weight loss may be more effective in increasing women’s participation in resistance training programs. However, more research is needed to determine the most effective ways to motivate men and women differently in resistance training interventions.

However, this study highlights the importance of considering gender differences in motivations for resistance training. While men and women exhibit similar exercise habits, their motivations may differ significantly. These findings may have important implications for resistance training interventions aimed at increasing participation and adherence in both men and women. Further research is needed to identify effective methods of motivating men and women in resistance training interventions. These results suggest that there are significant differences between men and women in terms of both the prevalence and consumption method of steroid use. Specifically, 53.05% of men and 41.99% of women reported using anabolic and androgenic steroids. This finding is significant as it highlights the need for gender-specific interventions to address steroid use. The size of the sample, participants, and gyms used in the literature varied considerably. For instance, a study conducted in Germany approximately 15 years ago involved 113 gyms and 621 individuals and reported a prevalence of AS use of 13.5% [14]. In Stockholm, Sweden, the prevalence was 3.8% with 64 gyms and 1746 individuals [19]. On the other hand, in Al-Ain, United Arab Emirates, the prevalence was 22.1% with 18 gyms and 154 individuals [20]. However, some studies had smaller sample sizes; for example, a study in El Paso, United States, evaluated three gyms and 516 individuals, revealing a prevalence of 11.0% [21]. Several factors, such as the sample distribution, the regional characteristics, and the individual characteristics of the samples, could have contributed to the variability in the prevalence of AS use among these studies. For instance, a study in the Netherlands that involved 92 gyms and 718 individuals reported a prevalence of AS use of 1% [22]. These findings are consistent with previous studies that have found that men are more likely to use anabolic and androgenic steroids than women [23–25]. The reason for lower consumption of AS among women is often due to their desire not to become excessively muscular or develop male characteristics [26]. On the other hand, men use AS not only to attain their desired body but also to gain status, admiration, and popularity in their social circle [27]. Furthermore, using AS helps them to be recognized and accepted by their peers [28]. These results also revealed important gender-based differences in the methods of steroid consumption, with 50.30% of men using intravenous injection and 49.05% of women using oral consumption. These differences may be due to various factors such as differences in physiology, availability, and perceived effectiveness. Of particular importance is the use of Testosterone by men and Winstrol by women, which were found to be the most commonly used steroids among the respective genders. While the reasons for these gender-based differences are unclear, they may reflect differences in physique ideals or perceived benefits or side effects.

### Strength and limitations

These findings have important implications for the development of interventions to address anabolic and androgenic steroid use. The fact that gender-based differences were found in both the prevalence and consumption method of steroid use highlights the need for gender-specific interventions that take into account the unique factors driving steroid use among men and women. For instance, interventions targeting men may need to focus on reducing intravenous injection use, while interventions targeting women may need to focus on reducing oral consumption. While this study provides valuable insights into gender-specific differences in anabolic and androgenic steroid use, it is important to note that the sample used in this study was limited to a specific population and may not be representative of the broader population. Additionally, self-reported data are subject to social desirability bias and may not reflect the true prevalence of anabolic and androgenic steroid use. Future studies should aim to replicate these findings with larger, more representative samples, and employ more objective measures of steroid use such as biological markers.

## Conclusion

In conclusion, our study significantly contributes to the understanding of resistance training practices among both genders, particularly among young adults. It underscores that resistance training is not limited to a specific demographic but is embraced by individuals with diverse educational and marital backgrounds. A key finding of our research is the distinct motivations for resistance training between men and women, with hypertrophy being a primary driver for men and weight loss for women. This divergence in motivations necessitates the development of gender-specific resistance training interventions to enhance participation and adherence. Furthermore, our study unveils critical gender differences in the prevalence and methods of anabolic steroid (AS) use. Men reported higher usage rates and a preference for intravenous injection, while women predominantly opted for oral consumption. These findings are pivotal, highlighting the need for gender-specific considerations when designing interventions and educational programs to address AS use among resistance training practitioners. Our research, therefore, provides valuable insights that can guide the development of more effective, gender-tailored strategies in the field of resistance training.

### Future studies

In future studies, several suggestions can be considered to enhance the straightness of research on anabolic steroid use among resistance training practitioners. First, adopting a longitudinal approach would provide valuable insights into the changes in steroid use over time post-pandemic, identifying shifts in prevalence, patterns, and influencing factors. Also, supplementing quantitative data with in-depth interviews would offer a deeper understanding of motivations, perceptions, and experiences related to steroid use. Moreover, comparing steroid use across different training settings, such as home-based workouts, commercial gyms, or community centers, would allow for a comparison of prevalence rates and factors associated with steroid use within these environments. Additionally, exploring psychological factors such as body image dissatisfaction, social pressure, or self-esteem would provide a more comprehensive understanding of the motivations behind steroid use. Lastly, investigating the effectiveness of educational initiatives aimed at raising awareness and assessing their impact on attitudes, knowledge, and behaviors related to steroid use would assist in designing evidence-based preventive strategies. Implementing these suggestions would contribute to a more comprehensive and robust understanding of anabolic steroid use among resistance training practitioners.

## Data Availability

The datasets used and/or analyzed during the current study are available from the corresponding author upon reasonable request.

## Abbreviations

AS: Anabolic Steroid
COVID-19: Coronavirus Disease 2019
